# Compassion Fatigue and Burnout Among Health Care Professionals: Protocol for a Scoping Review

**DOI:** 10.2196/66360

**Published:** 2025-07-23

**Authors:** Christian Guilherme Capobianco dos Santos, Martins Fideles dos Santos Neto, Stela Regina Pedroso Vilela Torres de Carvalho, Márcia Regina Furlani, Cíntia Canato Martins, Emerson Roberto Santos, João Daniel Souza Menezes, Matheus Querino da Silva, Loiane Letícia Santos, Thaysa Castro Molina, Natalia Almeida de Arnaldo Silva Rodriguez Castro, Helena Cristóvão., Randolfo Santos Júnior, Vânia MS Brienze, Alba Regina de Abreu Lima, Patricia da Fucuta, Denise Vaz-Oliani, Neide Aparecida Domingos, Maria Cristina Miyazaki, Gerardo Araújo Filho, Júlio César André

**Affiliations:** 1 School of Medicine of Sao Jose do Rio Preto Araçatuba Brazil; 2 Barretos Cancer Hospital São José do Rio Prêto Brazil; 3 School of Medicine of Sao Jose do Rio Preto São José do Rio Preto Brazil; 4 University of Santo Amaro - UNISA Sao Paulo Brazil

**Keywords:** burnout, compassion, compassion fatigue, compassion satisfaction, fatigue, health care professionals, health care workers, professional quality of life, scoping review protocol, scoping reviews, secondary traumatic stress, stress, traumatic stress

## Abstract

**Background:**

Compassion fatigue and burnout among health care professionals are growing concerns, impacting the well-being of both providers and patients.

**Objective:**

This scoping review aims to map existing evidence on the levels of compassion satisfaction, burnout, and secondary traumatic stress among health care professionals while identifying factors influencing their professional quality of life.

**Methods:**

We will conduct a scoping review using established methods proposed by Arksey and O’Malley and Levac et al, also incorporating the recommendations of the Joanna Briggs Institute for scoping reviews and reporting guidelines. EMBASE, ERIC, PubMed, Science Direct, Scopus, and Web of Science will be searched from March 2019 to March 2024, with an update closer to the time of manuscript submission. Gray literature sources will also be searched. Publications that contain primary studies, systematic reviews, meta-analyses, and clinical guidelines addressing compassion fatigue and burnout prevention in health care professionals will be selected for inclusion. Extracted data items will include study characteristics, interventions for the prevention of compassion fatigue and burnout, measures of compassion satisfaction, burnout, and secondary traumatic stress, as well as the quality of reporting and methodology.

**Results:**

A narrative synthesis with summary tables will be used to describe our findings. The review is expected to be completed by December 2025, and the search strategy has been developed and pilot-tested.

**Conclusions:**

Our findings will help identify gaps in the literature with respect to compassion fatigue and burnout prevention strategies for health care professionals. This review will provide a comprehensive overview of current research, informing future interventions and policies aimed at improving health care professionals’ well-being and job satisfaction.

**Trial Registration:**

Open Science Framework r83cu; https://osf.io/r83cu

**International Registered Report Identifier (IRRID):**

PRR1-10.2196/66360

## Introduction

### Overview

Compassion satisfaction among health care professionals is a crucial aspect of the quality of care provided. Compassion satisfaction refers to the positive feelings derived from caring for others and the ability to help effectively [1]. It is closely related to, yet distinct from, compassion fatigue, which can be defined as the emotional and physical exhaustion that health care workers can experience when exposed to prolonged periods of high emotional stress while caring for patients. These concepts are integral to understanding the professional quality of life of health care workers, which encompasses both positive and negative aspects of their work experience. When this satisfaction is absent, it can lead to significant negative consequences, such as burnout and secondary traumatic stress, compromising the effectiveness and quality of care offered to patients [2]. The professional quality of life of health care professionals is vital, as it directly affects their well-being and job satisfaction [3].

Recent studies have focused on examining compassion satisfaction, burnout, and secondary traumatic stress among health care professionals, revealing varying levels of these factors [3,4]. Research conducted in Brazil found that neonatal intensive care unit (ICU) nurses face higher levels of burnout compared to other ICU departments, indicating the specific challenges faced by health care professionals in this environment [5,6].

Various factors influence professional quality of life, including gender, sleep medication, education level, position, department, communication difficulty, and number of children [7,8]. These factors can impact professional quality of life in various ways. For instance, sleep medication use may indicate poor sleep quality, which can exacerbate stress and fatigue [9]. Education level and position may influence job responsibilities and autonomy, affecting job satisfaction [10,11]. Communication difficulties can lead to increased stress and potential conflicts [12]. The number of children may impact work-life balance [13]. Collectively, these factors can contribute to increased risk of burnout and decreased compassion satisfaction. Understanding these relationships is crucial for developing effective interventions to improve health care professionals’ well-being and, consequently, the quality of patient care. Therefore, health care organizations should focus on implementing strategies to promote better sleep hygiene and support the overall mental health and job satisfaction of their staff [7,8].

Informal and continuous education play an important role in preventing problems related to quality of life, suggesting that professional experience accumulated over the years can be a preventive factor [14,15]. Moreover, workload has a significant relationship with burnout and well-being, where an increase in workload can elevate the risk of burnout and, paradoxically, also be associated with well-being [13,14]. Compassion, satisfaction, and burnout are statistically significant predictors of well-being, with positive and negative relationships, respectively [14,15].

Health care professionals face emotional challenges when dealing with death and providing compassionate care, such as feelings of helplessness and sadness [16]. Ethical dilemmas and moral distress are prevalent, indicating the complexity of decision-making that these professionals face [17]. Psychological resilience and organizational support are fundamental for promoting well-being and reducing compassion fatigue [18,19].

The health care work relationship is strongly marked by the ambivalence between suffering and pleasure, with the physical and mental health of professionals often at risk [20]. Scientific production on moral distress among health care professionals showed significant growth between 2015 and 2018, with most articles published in English, reflecting a growing and global interest in the topic [20].

Health care professionals often face challenges when trying to align their care with their personal belief systems, resulting in acute moral distress [21]. This phenomenon is exacerbated by the lack of adequate resources and limited coping strategies to mitigate the intense demands of work [21]. Studies also reveal a high prevalence of stress among health care professionals, with a significant proportion reporting elevated levels of stress, anxiety, and exhaustion [22]. Continuous exposure to traumatic situations and painful patient stories contributes to compassion fatigue, known as vicarious traumatization [23].

To address these challenges, interventions such as increasing qualified staff, improving multidisciplinary collaboration, and promoting a healthy organizational culture have been suggested to mitigate moral distress and improve the well-being of health care professionals [24]. Furthermore, a healthy lifestyle, including regular physical activity, has demonstrated a correlation with lower burnout symptoms and better mental well-being among professionals [25].

### Objectives

The primary objectives of this scoping review are:

1. To map the existing evidence on levels of compassion satisfaction, burnout, and secondary traumatic stress among health care professionals across various health care settings.
2. To identify and analyze factors influencing the professional quality of life of health care workers, including workplace environment, workload, and organizational support.
3. To synthesize information on interventions and strategies aimed at preventing or mitigating compassion fatigue and burnout in health care professionals.
4. To identify gaps in the current literature regarding compassion fatigue and burnout prevention in health care settings, thereby informing future research directions.

### Justification

The scoping review is justified by the identification of substantial gaps in previous research, whose conclusions reverberate both in the theoretical and practical domains of the mental health field. The evidence highlighted the urgent need for comprehensive investigations addressing the prevalence of compassion fatigue among professionals in this area, as well as the need for a deeper understanding of the associated professional demands and resources [20]. Moreover, the growing consolidation of the concept of compassion fatigue signals the importance of considering the spiritual dimension in the context of mental health practices, suggesting a more in-depth analysis in this domain [21]. Therefore, the proposed scoping review aims to fill these gaps, promoting advances in scientific knowledge and providing essential subsidies for practice and the development of evidence-based interventions, aiming to improve the quality of care offered by mental health professionals.

## Methods

This scoping review will be conducted according to the methodological frameworks proposed by Arksey and O’Malley [26] and Levac et al [27], also incorporating the recommendations of the Joanna Briggs Institute (JBI) for scoping reviews. The review process will follow the following steps: (1) identification of the research question; (2) identification of relevant studies; (3) study selection; (4) data extraction; (5) synthesis and reporting of results; and (6) consultation with experts.

### Research Question

The research question guiding this scoping review is: “What has been published in the literature about compassion fatigue and coping with it among health care professionals in the workplace?”. This review will investigate both qualitative and quantitative studies to provide a comprehensive understanding of the topic. We will examine various measures and indicators of compassion fatigue, burnout, and compassion satisfaction levels among health care professionals. These may include standardized scales such as the Professional Quality of Life (ProQOL) scale, as well as qualitative descriptions of experiences and coping strategies. By including diverse study types and measurement approaches, we aim to capture the multifaceted nature of these phenomena in health care settings.

This question was established using the Population, Concept, Context (PCC) strategy, where the Population refers to health care professionals, the Concept is “compassion fatigue and coping,” and the Context encompasses “workplace.” The PCC strategy is widely used to formulate research questions in scoping and systematic reviews [28].

### Eligibility Criteria

The inclusion and exclusion criteria (Textbox 1) were defined based on the research question and the objective of the review, following the PCC framework:

1. Population: Health care professionals (including but not limited to doctors, nurses, allied health professionals, and mental health workers);
2. Concept: Compassion satisfaction, compassion fatigue, burnout, secondary traumatic stress, coping strategies, and interventions;
3. Context: Workplace settings in health care (hospitals, clinics, community health centers, etc).

Multimedia Appendix 1 summarizes the eligibility criteria for the scoping review.

**Inclusion and exclusion criteria.**

**Inclusion criteria**

- Primary studies, systematic reviews, meta-analyses, and clinical guidelines.
- Published between March 2019 and March 2024.
- In English, Portuguese, and Spanish.
- Addressing workplace prevention for compassion fatigue and burnout in health care professionals.
- Studies addressing compassion satisfaction, burnout, and secondary traumatic stress in health care professionals.

**Exclusion criteria**

- Studies that do not fit the scope of the review.
- Duplicate publications.
- Letters to the editor, editorials, and opinion articles.
- Studies that do not present empirical data or are not available in full-text.

### Information Sources

Searches will be conducted in the following electronic databases: EMBASE, ERIC, PubMed, Science Direct, Scopus, and Web of Science. In addition, a search for gray literature will be conducted, including theses, dissertations, government reports, and conference proceedings [29].

### Search Strategy

The search strategy will use descriptors in English, Portuguese, and Spanish, combined with Boolean operators (AND, OR, NOT). The search terms include “health care professionals,” “health workers,” “health team,” “burnout,” “compassion fatigue,” “compassion satisfaction,” “professional exhaustion,” “emotional exhaustion,” “occupational stress,” “burnout syndrome,” “impact,” “consequences,” “prevention,” “interventions,” “coping strategies,” “treatment,” “support,” “well-being,” “quality of life.” The search strategy will be adapted for each database to ensure comprehensive coverage.

Multimedia Appendix 2 shows the search strategy for the scoping review in the PubMed database.

### Study Selection

The study selection process will be performed by 2 reviewers independently, using the Covidence reference management software [30]. Initially, titles and abstracts will be screened, followed by full-text reading of potentially relevant studies. Discrepancies will be resolved by consensus or by a third reviewer. The selection process will follow the recommendations of PRISMA (Preferred Reporting Items for Systematic Reviews and Meta-Analyses) [31] and will be presented through a flowchart.

### Data Extraction

Data will be extracted using a standardized form, developed specifically for this review [32]. The form will be pilot-tested on a sample of studies to ensure its comprehensiveness and reliability. The form will include information on authorship, year of publication, country, objective, study design, population, outcomes assessed, main results, and conclusions. A total of 2 reviewers will independently extract data, with any discrepancies resolved through discussion or involvement of a third reviewer.

Multimedia Appendix 3 shows the data extraction form.

### Data Synthesis

Data synthesis and analysis data synthesis will be performed narratively, with tables and graphs to summarize the findings [33]. Qualitative analyses, such as thematic analysis, may be used to identify recurring themes in the included studies [34]. We will use Braun and Clarke’s (2006) framework for thematic analysis, which involves familiarization with the data, generating initial codes, searching for themes, reviewing themes, defining and naming themes, and producing the report [35]. Data synthesis will involve a narrative summary of the findings, organized thematically based on the research questions. To integrate the quantitative and qualitative data, we will use a mixed-methods approach. Quantitative data, such as prevalence rates of compassion fatigue and burnout, will be presented alongside qualitative themes to provide a more comprehensive understanding of the phenomenon. We will look for convergence, divergence, and complementarity between the quantitative and qualitative findings to answer our main research questions. Consultations with experts in the field of health care professional well-being and burnout prevention will be conducted to validate the findings and provide additional insights [27]. Expert feedback will be integrated into the final synthesis of results, contributing to the interpretation and contextualization of the findings.

### Protocol Registration

This scoping review adheres to the PRISMA-ScR (Preferred Reporting Items for Systematic Reviews and Meta-Analyses extension for Scoping Reviews) guidelines [36] to ensure transparency and quality of reporting (Multimedia Appendix 4). To further enhance transparency and reproducibility, the review protocol has been registered prospectively in the Open Science Framework (OSF) [37].

### Ethical Considerations

As this is a scoping review of existing literature, no ethical approval is required. However, we will ensure that all included studies have adhered to ethical standards in their conduct and reporting.

## Results

The search strategy will be finalized by July 2025, followed by the screening process in August and September 2025. Data extraction and analysis will occur in October and November 2025, with the final report being prepared in December 2025.

## Discussion

### Overview

This scoping review will provide a comprehensive overview of the current state of research on compassion fatigue, burnout, and secondary traumatic stress among health care professionals. The findings will allow us to map the levels of compassion satisfaction and its relationship with professional quality of life, focusing on the key factors that influence these outcomes, such as work environment, workload, and organizational support. In addition, interventions identified in the literature for the prevention of burnout and compassion fatigue will be summarized, offering valuable insights into potential strategies for improving health care professionals’ well-being.

We anticipate that the results will highlight important trends, including the prevalence of compassion fatigue and burnout across different health care settings, and the effectiveness of interventions aimed at mitigating these issues. These data will be instrumental in identifying critical gaps in the current literature, particularly in terms of understudied populations or regions where health care systems face unique challenges.

The findings are expected to show the need for targeted strategies that address health care professionals’ emotional resilience and coping mechanisms. These results may also inform the development of training programs and organizational policies aimed at reducing burnout, improving job satisfaction, and enhancing the overall mental health of health care workers.

### Principal Results

Based on existing literature and background research, it is anticipated that while compassion satisfaction plays a critical role in buffering against burnout and secondary traumatic stress, there remains a lack of consistent and effective interventions specifically tailored for health care professionals. The results underscore the need for more nuanced and individualized approaches that consider the specific contexts in which health care professionals operate, including factors such as the department they work in, their roles, and the intensity of their workload.

This scoping review will build upon existing literature by providing a comprehensive overview of compassion fatigue and burnout across various health care professions. Previous studies, such as those by [38,39], have focused on specific health care professions or settings. Our review aims to synthesize findings across the entire health care sector, potentially revealing patterns and insights that may not be apparent in more focused studies. In addition, by including both quantitative and qualitative studies, we hope to provide a more nuanced understanding of the experiences of health care professionals and the effectiveness of various coping strategies and interventions.

This scoping review is one of the first to synthesize the literature on compassion fatigue and burnout prevention strategies across various health care settings. The importance of promoting mental health and emotional resilience through workplace interventions, including peer support and mental health programs, will likely emerge as key themes.

### Limitations

One limitation of this review is its reliance on published literature from English, Portuguese, and Spanish, which may exclude studies from other languages that could contribute valuable insights. In addition, the quality of the studies included may vary, as the review does not plan to formally assess study quality, a common limitation in scoping reviews. Furthermore, the review will be confined to studies published between 2019 and 2024, which may not capture earlier works that provide foundational knowledge.

### Comparison with Prior Work

Compared to previous reviews [2], this study provides a more structured and comprehensive mapping of the factors affecting health care professionals’ professional quality of life. While earlier works [5] have focused on specific interventions or particular groups, such as nurses in neonatal ICUs, this review will offer a broader, cross-sectional analysis, covering diverse health care environments and professional roles. In addition, this review integrates findings from both primary research and gray literature, offering a more expansive view of the available evidence.

### Implications for Practice and Research

The results of this scoping review will have significant implications for health care organizations, educational institutions, and policy makers. The identified strategies for preventing compassion fatigue and burnout can inform the development of targeted interventions and workplace policies. For researchers, this review will highlight critical gaps in the current literature, suggesting avenues for future studies, particularly in understudied health care settings or professional groups.

### Conclusions

The results of this scoping review will provide essential insights into compassion fatigue and burnout prevention strategies, helping to inform future interventions and policies. These findings will be valuable for health care organizations, educational institutions, and policy makers aiming to improve health care professionals’ well-being and job satisfaction. The review will also contribute to the growing body of literature on mental health in the health care workforce, offering new avenues for research and practical solutions to address these critical challenges.

By identifying key gaps in the current literature, this review will guide future research aimed at enhancing the mental resilience of health care professionals, ultimately improving the quality of care they provide to patients.

### Dissemination of Results

The findings of this scoping review will be disseminated through publication in a peer-reviewed journal and presentation at relevant conferences. We will also create a summary of the findings for stakeholders, including health care administrators and policy makers, to facilitate the translation of research into practice.

## Appendix

Multimedia Appendix 1 Eligibility criteria for the scoping review.

Multimedia Appendix 2 Search strategy for the scoping review in PubMed database.

Multimedia Appendix 3 Data extraction form.

Multimedia Appendix 4 PRISMA-P (Preferred Reporting Items for Systematic Review and Meta-Analysis Protocols) checklist.

## Abbreviations

ICU: intensive care unit
JBI: Joanna Briggs Institute
OSF: Open Science Framework
PCC: Population, Concept, Context
PRISMA: Preferred Reporting Items for Systematic Reviews and Meta-Analyses
PRISMA-ScR: Preferred Reporting Items for Systematic Reviews and Meta-Analyses extension for Scoping Reviews
ProQOL: Professional Quality of Life

## Data Availability

Data sharing is not applicable to this article as no new datasets were generated or analyzed during this study.
