# Supplementary material for: Compassion Fatigue and Burnout Among Health Care Professionals: Protocol for a Scoping Review
Source: JMIR Res Protoc. 2025 Jul 23;14:e66360. doi: 10.2196/66360 (PMC12329385; doi:10.2196/66360)
Supplement: Multimedia Appendix 1 [file resprot_v14i1e66360_app1.docx]

**Table S1.**

| Inclusion criteria | Exclusion criteria |
| --- | --- |
| Primary studies, systematic reviews, meta-analyses, and clinical guidelines  Published between March 2019 and March 2024  In English, Portuguese, and Spanish  Addressing workplace prevention for compassion fatigue and burnout in healthcare professionals  Studies addressing compassion satisfaction, burnout, and secondary traumatic stress in healthcare professionals | Studies that do not fit the scope of the review  Duplicate publications  Letters to the editor, editorials, and opinion articles  Studies that do not present empirical data or are not available in full text  Source: Author |
