# Supplementary material for: Compassion Fatigue and Burnout Among Health Care Professionals: Protocol for a Scoping Review
Source: JMIR Res Protoc. 2025 Jul 23;14:e66360. doi: 10.2196/66360 (PMC12329385; doi:10.2196/66360)
Supplement: Multimedia Appendix 2 [file resprot_v14i1e66360_app2.docx]

**Table S1.**

| Database | Search Strategy |
| --- | --- |
| PubMed | (“Compassion Fatigue”[Title/Abstract] OR “Fatigue, Compassion”[Title/Abstract] OR "compassion satisfaction"[Title/Abstract] OR “Secondary Trauma”[Title/Abstract] OR “Secondary Traumas”[Title/Abstract] OR “Trauma, Secondary”[Title/Abstract] OR “Traumas, Secondary”[Title/Abstract] OR “Secondary Traumatization”[Title/Abstract] OR “Secondary Traumatizations”[Title/Abstract] OR “Traumatization, Secondary”[Title/Abstract] OR “Traumatizations, Secondary”[Title/Abstract] OR “Vicarious Trauma”[Title/Abstract] OR “Traumas, Vicarious”[Title/Abstract] OR “Trauma, Vicarious”[Title/Abstract] OR “Vicarious Traumas”[Title/Abstract] OR “Secondary Traumatic Stress”[Title/Abstract] OR “Stresses, Secondary Traumatic”[Title/Abstract] OR “Stress, Secondary Traumatic”[Title/Abstract] OR “Traumatic Stress, Secondary”[Title/Abstract] OR “Vicarious Traumatization”[Title/Abstract] OR “Traumatization, Vicarious”[Title/Abstract] OR “Burnout, Psychological”[Title/Abstract] OR “Psychological Burnout”[Title/Abstract] OR “Burn-out”[Title/Abstract] OR “Burn out”[Title/Abstract] OR “Psychological Burn-out”[Title/Abstract] OR “Burn-out, Psychological”[Title/Abstract] OR “Psychological Burn out”[Title/Abstract] OR “Burnout”[Title/Abstract] OR “Burnout Syndrome”[Title/Abstract] OR “Burn-out Syndrome”[Title/Abstract] OR “Burn out Syndrome”[Title/Abstract] OR “Burnout, Student”[Title/Abstract] OR “Burnout, School”[Title/Abstract] OR “School Burnout”[Title/Abstract] OR “Student Burnout”[Title/Abstract]) AND (“Prevention and Control”[Title/Abstract] OR “Preventive Measures”[Title/Abstract] OR “Preventive Therapy”[Title/Abstract] OR “Prophylaxis”[Title/Abstract] OR “Control”[Title/Abstract] OR “Prevention”[Title/Abstract]) AND (“Workplace”[Title/Abstract] OR “Workplaces”[Title/Abstract] OR “Job Site”[Title/Abstract] OR “Job Sites”[Title/Abstract] OR “Work Location”[Title/Abstract] OR “Location, Work”[Title/Abstract] OR “Work Locations”[Title/Abstract] OR “Worksite”[Title/Abstract] OR “Worksites”[Title/Abstract] OR “Work Place”[Title/Abstract] OR “Work Places”[Title/Abstract] OR “Work-Site”[Title/Abstract] OR “Work Site”[Title/Abstract] OR “Work-Sites”[Title/Abstract]) AND (“Health Personnel”[Title/Abstract] OR “health professionals”[Title/Abstract] OR “health professional”[Title/Abstract] OR “Personnel, Health”[Title/Abstract] OR “Healthcare Workers”[Title/Abstract] OR “Healthcare Worker”[Title/Abstract] OR “Health Care Providers”[Title/Abstract] OR “Health Care Provider”[Title/Abstract] OR “Provider, Health Care”[Title/Abstract] OR “Healthcare Providers”[Title/Abstract] OR “Healthcare Provider”[Title/Abstract] OR “Provider, Healthcare”[Title/Abstract] OR “Health Care Professionals”[Title/Abstract] OR “Health Care Professional”[Title/Abstract] OR “Professional, Health Care”[Title/Abstract]) |
