# Supplementary material for: Compassion Fatigue and Burnout Among Health Care Professionals: Protocol for a Scoping Review
Source: JMIR Res Protoc. 2025 Jul 23;14:e66360. doi: 10.2196/66360 (PMC12329385; doi:10.2196/66360)
Supplement: Multimedia Appendix 3 [file resprot_v14i1e66360_app3.docx]

**Table S1.**

| 1. | General Information Study ID |  |
| --- | --- | --- |
| 2. | Authors |  |
| 3. | Year of Publication |  |
| 4. | Country |  |
| 5. | Type of Publication: (Primary Study / Systematic Review / Meta-Analysis / Clinical Guideline) Study Characteristics |  |
| 6. | Study Objective |  |
| 7. | Study Design |  |
| 8. | Study Population |  |
| 9. | Sample Size |  |
| 10. | Work Environment |  |
| 11. | Interventions and Outcomes Interventions for Prevention of Compassion Fatigue and Burnout |  |
| 12. | Measures of Compassion Satisfaction |  |
| 13. | Measures of Burnout |  |
| 14. | Measures of Secondary Traumatic Stress |  |
| 15. | Other Relevant Outcomes |  |
| 16. | Main Results Main Findings on Compassion Satisfaction |  |
| 17. | Main Findings on Burnout |  |
| 18. | Main Findings on Secondary Traumatic Stress |  |
| 19. | Other Relevant Results |  |
| 20. | Conclusions and Implications Main Conclusions |  |
| 21. | Implications for Practice |  |
| 22. | Implications for Future Research |  |
| 23. | Quality Assessment Methodological Quality (High / Medium / Low): Study Limitations |  |
| 24. | Additional Information Funding |  |
| 25. | Conflicts of Interest |  |
| 26. | Reviewers’ Comments |  |
