# Supplementary material for: Compassion Fatigue and Burnout Among Health Care Professionals: Protocol for a Scoping Review
Source: JMIR Res Protoc. 2025 Jul 23;14:e66360. doi: 10.2196/66360 (PMC12329385; doi:10.2196/66360)
Supplement: Multimedia Appendix 4 [file resprot_v14i1e66360_app4.docx]

**Table S1.**

| **SECTION** | **ITEM** | **PRISMA-ScR CHECKLIST ITEM** | **REPORTED ON PAGE #** |
| --- | --- | --- | --- |
| **TITLE** | | | |
| Title | 1 | Identify the report as a scoping review. | Y.  The title of the manuscript identifies the study as a “Scoping Review Protocol” |
| **ABSTRACT** | | | |
| Structured summary | 2 | Provide a structured summary that includes (as applicable): background, objectives, eligibility criteria, sources of evidence, charting methods, results, and conclusions that relate to the review questions and objectives. | Y  The Abstract provides a structured summary, including:  - Background: Mentions the growing concern about compassion fatigue and burnout among healthcare professionals.  - Objective: To map the existing evidence on levels of compassion satisfaction, burnout and secondary traumatic stress.  - Methods: Describes the methodology of the scoping review.  - Results: Indicates that a narrative synthesis with summary tables will be used.  - Conclusions: Points out that the results will help to identify gaps in the literature. |
| **INTRODUCTION** | | | |
| Rationale | 3 | Describe the rationale for the review in the context of what is already known. Explain why the review questions/objectives lend themselves to a scoping review approach. | Y  The Introduction describes the importance of compassion satisfaction and the negative impacts of its absence, such as burnout and secondary traumatic stress. It explains the need to investigate these factors in order to improve the well-being of healthcare professionals and the quality of care. |
| Objectives | 4 | Provide an explicit statement of the questions and objectives being addressed with reference to their key elements (e.g., population or participants, concepts, and context) or other relevant key elements used to conceptualize the review questions and/or objectives. | Y  The objectives are clearly defined in the “Objectives” section:  - To map the evidence on levels of compassion satisfaction, burnout and secondary traumatic stress.  - To analyze factors that influence quality of working life.  - Synthesize information on interventions and prevention strategies.  - To identify gaps in the literature. |
| **METHODS** | | | |
| Protocol and registration | 5 | Indicate whether a review protocol exists; state if and where it can be accessed (e.g., a Web address); and if available, provide registration information, including the registration number. | Y  The “Protocol registration” section indicates that the scoping review protocol will be registered with the Open Science Framework (OSF).  DOI 10.17605/OSF.IO/R83CU |
| Eligibility criteria | 6 | Specify characteristics of the sources of evidence used as eligibility criteria (e.g., years considered, language, and publication status), and provide a rationale. | Y  The "Eligibility Criteria" section specifies the characteristics of the evidence sources, including:  - Study types: Primary studies, systematic reviews, meta-analyses, and clinical guidelines.  - Period: Published between March 2019 and March 2024.  - Languages: English, Portuguese, and Spanish.  - Focus: Addressing workplace prevention for compassion fatigue and burnout in healthcare professionals. |
| Information sources* | 7 | Describe all information sources in the search (e.g., databases with dates of coverage and contact with authors to identify additional sources), as well as the date the most recent search was executed. | Y  The "Information Sources" section lists the electronic databases to be searched: EMBASE, ERIC, PubMed, Science Direct, Scopus, and Web of Science. It also mentions the search for gray literature. |
| Search | 8 | Present the full electronic search strategy for at least 1 database, including any limits used, such that it could be repeated. | Y  The "Search Strategy" section describes the use of descriptors in English, Portuguese, and Spanish, combined with Boolean operators. Multimedia Appendix 2 shows the search strategy for the PUBMED database. |
| Selection of sources of evidence† | 9 | State the process for selecting sources of evidence (i.e., screening and eligibility) included in the scoping review. | Y  The "Study Selection" section describes the selection process, which will be performed independently by two reviewers using Covidence software. It includes screening of titles and abstracts, followed by full-text reading. |
| Data charting process‡ | 10 | Describe the methods of charting data from the included sources of evidence (e.g., calibrated forms or forms that have been tested by the team before their use, and whether data charting was done independently or in duplicate) and any processes for obtaining and confirming data from investigators. | Y  The "Data Extraction" section describes the use of a standardized form for data extraction, which will be pilot-tested to ensure comprehensiveness and reliability. Extraction will be done independently by two reviewers. Appendix 3 shows the data extraction form. |
| Data items | 11 | List and define all variables for which data were sought and any assumptions and simplifications made. | Y  The "Data Extraction" section mentions that the form will include information on authorship, year of publication, country, objective, study design, population, outcomes assessed, main results, and conclusions. |
| Critical appraisal of individual sources of evidence§ | 12 | If done, provide a rationale for conducting a critical appraisal of included sources of evidence; describe the methods used and how this information was used in any data synthesis (if appropriate). | N/A  The manuscript does not detail conducting a critical appraisal of the included evidence sources. |
| Synthesis of results | 13 | Describe the methods of handling and summarizing the data that were charted. | Y  The "Data synthesis" section describes that data synthesis will be performed narratively, with tables and graphs to summarize the findings. Qualitative analyses, such as thematic analysis, may be employed. |
| **RESULTS** | | | |
| Selection of sources of evidence | 14 | Give numbers of sources of evidence screened, assessed for eligibility, and included in the review, with reasons for exclusions at each stage, ideally using a flow diagram. | N/A  This section will be completed upon conclusion of the review, with the numbers of evidence sources screened, assessed, and included. |
| Characteristics of sources of evidence | 15 | For each source of evidence, present characteristics for which data were charted and provide the citations. | N/A  This section will be completed upon conclusion of the review, with the characteristics of the evidence sources and citations. |
| Critical appraisal within sources of evidence | 16 | If done, present data on critical appraisal of included sources of evidence (see item 12). | N/A  As there is no detailed critical appraisal, this section remains N/A. |
| Results of individual sources of evidence | 17 | For each included source of evidence, present the relevant data that were charted that relate to the review questions and objectives. | N/A  This section will be completed upon conclusion of the review, with the relevant data extracted from each evidence source. |
| Synthesis of results | 18 | Summarize and/or present the charting results as they relate to the review questions and objectives. | N/A  This section will be completed upon conclusion of the review, with a summary of the synthesis results. |
| **DISCUSSION** | | | |
| Summary of evidence | 19 | Summarize the main results (including an overview of concepts, themes, and types of evidence available), link to the review questions and objectives, and consider the relevance to key groups. | N/A  This section will be completed upon conclusion of the review, with a summary of the main results and their relevance. |
| Limitations | 20 | Discuss the limitations of the scoping review process. | Y  The "Limitations" section discusses the limitations of the scoping review process, such as reliance on published literature in English, Portuguese, and Spanish, and the lack of formal assessment of study quality. |
| Conclusions | 21 | Provide a general interpretation of the results with respect to the review questions and objectives, as well as potential implications and/or next steps. | N/A  This section will be completed upon conclusion of the review, with a general interpretation of the results and implications. |
| **FUNDING** | | | |
| Funding | 22 | Describe sources of funding for the included sources of evidence, as well as sources of funding for the scoping review. Describe the role of the funders of the scoping review. | Y  The "Funding Information" section indicates that the study did not receive specific funding from funding agencies or external sources. |
